# Longitudinal study of cross-reactive antigenemia in individuals with high *Loa loa* microfilarial density reveals promising biomarkers for distinguishing lymphatic filariasis from loiasis

**DOI:** 10.3389/fpara.2023.1292837

**Published:** 2023-11-16

**Authors:** Linda Djune-Yemeli, Marla Hertz, Hugues C. Nana-Djeunga, Amy Rush, Petra Erdmann-Gilmore, Robert Sprung, Jean Gabin Bopda, Reid Townsend, Palmer Masumbe Netongo, Joseph Kamgno, Philip J. Budge

**Affiliations:** 1 Higher Institute of Scientific and Medical Research (ISM), Yaoundé, Cameroon; 2 Molecular Diagnostics Research Group, Biotechnology Centre-University of Yaoundé I (BTC-UY-I), Yaoundé, Cameroon; 3 Office of Scholarly Communication, University of Alabama at Birmingham Libraries, Birmingham, AL, United States; 4 Infectious Disease Division, Department of Medicine, Washington University in St. Louis, St. Louis, MO, United States; 5 Division of Endocrinology, Metabolism and Lipid Research, Department of Medicine, Washington University School of Medicine, St. Louis, MO, United States; 6 Departement of Biochemistry, Faculty of Sciences, University of Yaoundé I, Yaoundé, Cameroon; 7 Faculty of Medicine and Biomedical Sciences, University of Yaoundé I, Yaoundé, Cameroon

**Keywords:** Loiasis, lymphatic filariasis, cross-reactivity, LF-rapid diagnostic tests, biomarker

## Abstract

**Background and methods:**

Circulating *Loa loa* antigens are often detected in individuals with heavy *L. loa* infections by diagnostic tests for lymphatic filariasis (LF) caused by *Wuchereria bancrofti*. This is a major challenge to LF mapping and elimination efforts in loiasis co-endemic areas. However, it also provides an opportunity to identify antigen biomarkers for loiasis. To determine which *L. loa* antigens might be promising biomarkers for distinguishing true LF from loiasis, we screened for *L. loa* antigens in a group of individuals with heavy *L. loa* infections living in the Okola Health District of Cameroon. In this longitudinal study, participants were tested for cross-reactive antigenemia by filariasis test strip (FTS), ELISA, and western blot, and were monitored for FTS status at 6, 9, 12, and 15 months post-enrollment. We then identified specific circulating *L. loa* antigens by liquid chromatography-tandem mass spectrometry (LC-MS/MS) from baseline and 15-month plasma samples.

**Principal findings and conclusions:**

Among 73 FTS-positive (FTS+) and 13 FTS-negative (FTS−) participants with high *L. loa* microfilarial loads, 83% maintained their FTS status over the course of the study, while 17% experienced at least one FTS conversion event (from FTS+ to FTS− or vice versa). Cross-reactive antigens were detected in both FTS+ and FTS− sera by western blot, and there was poor agreement in antigen detection by FTS, western blot, and ELISA methods. One protein family, a group of Nas-14 metalloproteases, was detected by LC MS/MS in >80% of tested samples, including FTS− samples. These data identify Nas-14 as a promising loiasis biomarker potentially capable of distinguishing loiasis from lymphatic filariasis.

## 1 Introduction

Lymphatic filariasis (LF) is a debilitating, mosquito-borne disease characterized by chronic morbidity in the form of hydrocele, lymphedema, and elephantiasis (Herrera et al., 2011; World Health Organization, 2019). Recognizing its important socioeconomic impact, and with advances in diagnostics and treatment, the World Health Organization (WHO) launched the Global Program to Eliminate LF (GPELF) in 2000 (Ottesen, 2000). GPELF’s elimination strategy has 2 pillars: (i) interrupting parasite transmission through annual mass drug administration (MDA), and (ii) offering a basic package of care to alleviate the suffering of those affected with elephantiasis/hydrocele (World Health Organization, 2010). Although considerable progress has been made toward LF elimination, significant efforts are still needed to meet the elimination target that has now been postponed to 2030 (World Health Organization, 2021).

To achieve LF elimination, one of the critical actions recommended in WHO’s 2021–2030 roadmap is to implement MDA in all LF-endemic implementation units (NTD Modelling Consortium Lymphatic Filariasis Group, 2019; World Health Organization, 2021). However, in some settings in West and Central Africa, loiasis is an obstacle to LF elimination for two reasons. First, individuals with heavy *L. loa* infections are at high risk for serious adverse events if treated during MDA (Gardon et al., 1997). Second, LF mapping, the crucial first step in MDA implementation (Zouré et al., 2011; Kelly-Hope et al., 2017; Deribe et al., 2020), can be inaccurate in loiasis-endemic areas due to cross-reactive antigenemia. LF mapping mainly relies on detection of a *W. bancrofti*-specific circulating filarial antigen (CFA) by rapid diagnostic test (RDT), most commonly the Filariasis Test Strip (FTS) (Lindsay and Thomas, 2000; Ruberanziza et al., 2009; Rebollo and Bockarie, 2013; Mwase et al., 2014). Although LF RDTs have supported programmatic activities in West and Central Africa for decades (Rocha et al., 2009; Weil et al., 2013; Chesnais et al., 2017), there is now strong evidence that they lack specificity in loiasis co-endemic areas due to the presence of cross-reactive circulating *L. loa* antigens in some individuals heavily infected with loiasis (Bakajika et al., 2014; Wanji et al., 2015; Pion et al., 2016; Wanji et al., 2016; Hertz et al., 2018; Wanji et al., 2019). While loiasis cross-reactivity is more likely in individuals with high *Loa loa* microfilaria (Mf) counts (> 20,000 mf/mL), it is not well understood why only some heavily infected individuals test positive by FTS, or why some FTS-positive individuals may revert to a negative FTS despite maintaining high Mf loads (Hertz et al., 2018).

Thus, loiasis cross-reactivity makes it near impossible for national programs in loiasis endemic areas to determine with certainty where LF MDA is required and when it should be stopped. The prevalence of loiasis cross-reactivity can easily exceed 6% in high loiasis endemic settings (Bakajika et al., 2014), far exceeding the 1% threshold for implementing LF MDA (World Health Organization, 2019). Although potential target antigens have been described (Drame et al., 2016), no antigen detection assays for loiasis are currently available for programmatic use. The development of a rapid antigen detection test for loiasis to confirm whether positive FTS results were due to the presence of a *L. loa* antigen would improve mapping accuracy of both infections in co-endemic areas. The purposes of this study were to investigate the stability of loiasis cross-reactivity over time and to determine which of the hundreds of *L. loa* proteins previously detected in cross-reactive plasma (Hertz et al., 2018) are consistently present in cross-reactive sera and can therefore be used to distinguish LF from loiasis.

## 2 Materials and methods

### 2.1 Ethics statement

The current study received ethical approval from the Regional Ethics Committee for Human Health Research, Centre Region, Cameroun (CE № 05862/CRERSHC/2019) and the Institutional Review Board of the Washington University in Saint Louis (IRB ID #: 01909003). In addition, administrative authorization was granted by the Okola District Medical Officer. Prior to the beginning of the surveys, the objectives and schedules of the study were explained to all the participants and written informed consents obtained.

### 2.2 Study area

This study was conducted in the Okola Health District (HD), Centre Region, Cameroon, which is highly endemic for loiasis and hypo- to meso-endemic for onchocerciasis, with no evidence of Bancroftian filariasis (Zouré et al., 2011; Kouam et al., 2013). Ivermectin MDA was initiated in the 11 health areas of the Okola in 1999. However, due to the occurrence of several adverse event cases, MDA activities were stopped. The situation was clarified (identification of loiasis as main risk factor and elaboration of mitigating measures) and MDA resumed in 5 of the 11 health areas considered meso-endemic for onchocerciasis. A Test and Not Treat (TaNT) strategy was piloted in the Okola HD in 2015 (Kamgno et al., 2017) to safely extend ivermectin-based MDA in the 6 remaining health areas hypo-endemic to onchocerciasis (World Health Organization, 2007). Okola HD is a degraded forest area, with a population estimated at 54,870 inhabitants (Ministry of Public Health, 2017). Agriculture (crops, cacao, and nut) and trading are the main activities in the area.

### 2.3 Study design

This was a longitudinal study involving a cohort of adults (age ≥18 years) heavily infected with *L. loa* (the choice of heavily infected individuals was guided by the strong association between high *L. loa* Mf density and false-positive LF rapid test results (Pion et al., 2016). Individuals excluded from the TaNT pilot study in the Okola HD due to *L. loa* Mf counts higher than 20,000 Mf/mL (Kamgno et al., 2017) were screened in January and February 2020. Daytime capillary blood was collected by fingerpick and tested with FTS (Abbott Bioline) and for *L. loa* Mf density by LoaScope (D’Ambrosio et al., 2015). FTS-positive individuals were enrolled in the study as cases, and a subset of heavily infected FTS-individuals were also enrolled as controls. After enrolment, participants provided daytime capillary and venous blood for calibrated thick blood smears, ELISA and proteomic analyses, and nighttime capillary blood for *W. bancrofti* thick blood smear and PCR.

**FIGURE 1:**
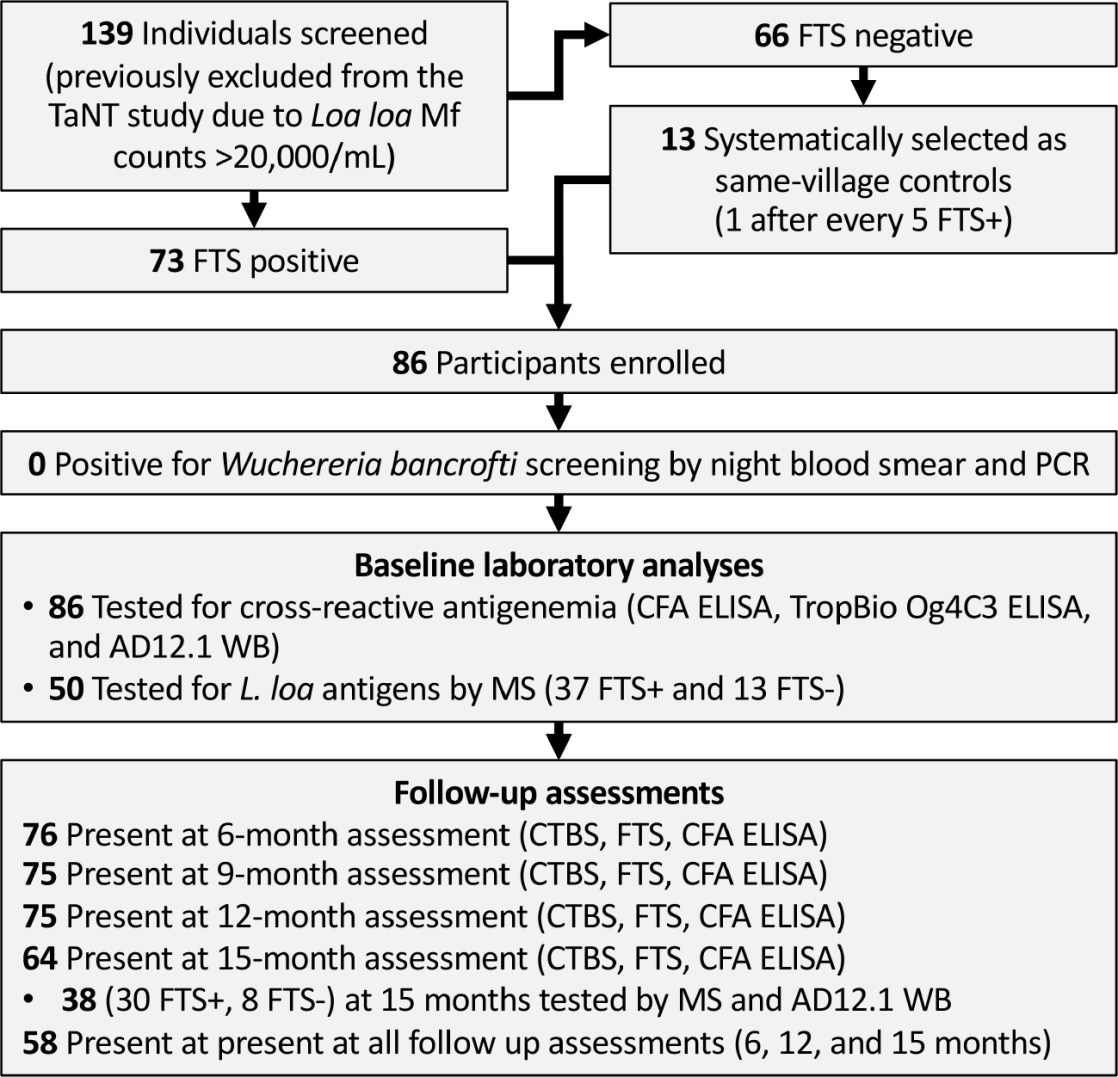
Flow chart showing field study, sample collection and analyses. TaNT, Test and Not Treat; FTS, Filariasis Test Strip; CFA, circulating filarial antigen; WB, western blot; MS, mass spectrometry.

We intended to assess persistence of cross-reactive antigenemia quarterly over the course of one year. However, the assessment planned at 3 months post-enrolment was not possible due to the COVID-19 pandemic. We therefore tested participants at baseline, 6-, 9-, 12-, and 15-months post-enrolment. At each follow-up visit, daytime capillary blood was collected and tested for Mf and *L. loa* cross-reactive antigen (by FTS), and venous blood was collected for subsequent ELISA and proteomic analyses (Figure 1). For proteomics analyses, endemic and non-endemic control were selected among banked sera from previous studies; endemic controls were amicrofilaremic patients in the Akonolinga and Awae health district, Centre Cameroon (Hertz et al., 2018), while non-endemic controls were de-identified clinical samples collected at Barnes-Jewish Hospital, St Louis Missouri, US, from patients who have never been in loiasis endemic areas.

### 2.4 Sample size

The objective of this study was to identify one or more antigens persistently present among those with cross-reactive loiasis, that can be used as biomarker(s) to distinguish cross-reactive loiasis from LF. We define a potential biomarker as an antigen that is detectable in at least 90% of the population of interest. Therefore, a minimal sample size of 50 participants (for mass spectrometry analyses) was sufficient to give us 95% confidence in excluding antigens that are present in less than 80% of samples (1-sided Wilson score lower than the 95% confidence interval).

### 2.5 LoaScope and calibrated thick blood smear (CTBS)

Blood for *L. loa* Mf density measurement was collected between 10:00–16:00 and was assessed by LoaScope as previously described (D’Ambrosio et al., 2015), and by calibrated thick blood smear (CTBS). Enrolled participants also underwent nocturnal (between 22:00–02:00) blood collection for CTBS to rule out *W. bancrofti*. For each CTBS, 70 μL of non-heparinized finger-prick blood was collected using calibrated capillary tube and ~50 μL was spread onto a microscope slide. The slide was then allowed to air dry and was stained with 10% Giemsa using standard procedures (Sasa, 1976). Giemsa-stained smears were examined under a light microscope at 100X magnification for species identification and quantitation of blood dwelling Mf.

### 2.6 DNA extraction and qPCR for *W. bancrofti* and *L. loa*

Nocturnal blood was also tested for *W. bancrofti* and *L. loa* DNA by quantitative PCR as previously described (Bakajika et al., 2014). Fingerpick blood was collected on calibrated filter paper discs (TropBio, Cellabs Pty Ltd, NSW, Australia) and allowed to air dry. Three of the six-disc protrusions (calibrated to hold 10 uL each) of each dried blood spot (DBS), equivalent to ~30 μL whole blood, were used for DNA extraction by QIAamp DNA mini kits (Qiagen, Valencia, CA). Quantitative PCR using Taqman Multiplex master mix (Applied Biosystems, Foster City, CA) was performed with an ABI Quant 6 instrument under standard conditions using *W. bancrofti* and *L. loa* specific primers and probes as previously described (Rao et al., 2006; Fink et al., 2011).

### 2.7 Detection and quantification of cross-reactive antigens

*L. loa* cross-reactive antigens were detected/quantified in this study using several LF assays: the rapid diagnostic test FTS, the TropBio Og4C3 ELISA, the in-house (IH) sandwich ELISA, and immunoprecipitative western blot. Details on the antibodies used in the different assays are provided in Table 1.

#### 2.7.1 Bioline Filariasis test strip (FTS)

Cross-reactive loiasis antigens were detected by FTS (Abbott Diagnostics, Scarborough, Maine, USA) as per manufacturer’s recommendations. The test results were read at 10 min and recorded according to a grading procedure described by Chesnais et al. (2013). Negative tests (no visible test line) were scored as zero, FTS with a clearly visible test line weaker than the control line were scored as 1, those with test lines of similar intensity as the control line were scored as 2, and those with test lines darker than the control lines were scored as 3.

#### 2.7.2 In-house CFA ELISA

Detection of *L. Loa* cross-reactive antigens by sandwich ELISA was performed as previously described, using two analog monoclonal antibodies (AD12.1 and DH6.5) that recognize the same carbohydrate moiety (Weil et al., 1987; Hertz et al., 2018). Briefly, plates were coated with monoclonal antibody DH6.5 at 20 μg/mL in 0.01 M carbonate buffer, pH 8, and incubated overnight at 37°C. Wells were washed with phosphate-buffered saline (PBS) containing 0.05% Tween-20 (PBS-T) and subsequently blocked with 200 μL of 5% fetal calf serum in PBS-T for one hour at 37° C. Human sera were mixed 1:1 with 0.1M EDTA and heated at 56°C for 30 min to dissociate immune complexes (Weil and Liftis, 1987). Fifty microliters of treated sera were then added to the wells and incubated for two hours at 37°C. Wells were washed 3 times with PBS-T and incubated with HRP-conjugated monoclonal antibody AD12.1 for one hour at 37°C. After a final round of three PBS-T washes, 100 μL O-phenylenediamine dihydrochloride (OPD, Thermo Fisher Scientific Inc., Waltham, Massachusetts, USA) substrate was added and allowed to develop for 15 minutes. The reaction was stopped by adding 30 μL of 4 M sulfuric acid, and absorbance was read at 492nm. Cross-reactive antigen levels were determined by automated fit to a calibration curve standard. An antigen value ≥ 6 ng/mL was considered as positive for the CFA ELISA assay.

#### 2.7.3 TropBio Og4C3 ELISA

The TropBio Og4C3 ELISA test kit was used following the manufacturer (TropBio Pty ldt, Queensland, Australia) instructions. Briefly, 100 mL of each individual plasma sample was mixed with 300 μL EDTA solution and boiled at 100°C for 5 min to release the heat stable CFA in positive specimens. After centrifugation (10,000 rpm for 5 min), 50 μL of supernatant was added, in duplicate, to plates previously precoated with the monoclonal anti-filarial antibody Og4C3. Serial dilutions of the supplied positive and negative controls were included in duplicates. After overnight incubation, plates were washed as before then incubated for 1 hour at room temperature with 50 μL of rabbit anti-CFA antibody. After a final washing, the plate was incubated for one hour at room temperature with 50 μmL of anti-rabbit horse-radish-peroxidase-conjugate. Plates were developed by addition of the supplied substrate, and the optical density (OD) was read at 450 nm. An ELISA signal ≥ 0.2 was considered positive.

### 2.8 Immunoprecipitation of cross-reactive antigens and analyses by western blot and mass spectrometry

To capture cross-reactive antigen, we immunoprecipitated circulating filarial antigens as previously described (Hertz et al., 2018). Affigel-10 beads (Bio-Rad, Hercules, California, United States) were conjugated to monoclonal DH6.5 according to the manufacturer’s protocol. Fifty microliters of DH6.5-conjugated beads were mixed with 1mL of human sera and incubated overnight with rocking at 4°C. The next morning, the beads were washed four times with Thermo wash buffer (15mM NaCl, 1mM EDTA, 25mM Tris pH 7.4, 1% NP-40 and 5% glycerol), then twice in cold PBS to remove the detergent. After washing, beads were suspended in 1X NuPAGE LDS sample buffer (Invitrogen Inc, Carlsbad, California, USA) and incubated at 95°C for 5 min to release bound antigen. Immunoprecipitation products were assayed by western blot and mass spectrometry.

For western blot, proteins eluted from DH6.5 conjugated beads were separated by SDS-PAGE using a 4–12% bis-tris NuPAGE gels (Invitrogen Inc, Carlsbad, California, USA) and transferred to nitrocellulose membranes (Amersham BioSciences, Buckinghamshire, UK). Blots were incubated with blocking buffer [5% milk in phosphate-buffered saline with 0.05% Tween-20 (PBS-T)] for one hour, then incubated for one hour at room temperature with HRP-conjugated AD12.1.1 antibody, diluted 1:3000 in blocking buffer. Membranes were washed three times in PBS-T and incubated with Clarity Western ECL substrate (Bio-Rad, Hercules, California, United States) for chemiluminescence detection using an Azure c600 imager.

Immunoprecipitated samples were prepared for LC-MS as previously described (Hertz et al., 2018) and analyzed by mass spectrometry using a TimsTOF PRO spectrometer coupled to a nanoElute LC system (Bruker) or a Q-Exactive coupled to an Easy-nano-LC1000 (Thermo Scientific). Data from the mass spectrometer were converted to peak lists and the MS2 spectra were analyzed using Peaks software (Ma et al., 2003) (Bioinformatics Solutions, Waterloo, Ontario Canada; Peaks Studio version 10.6). Peaks was set up to search against a custom database of *L. loa* proteins (12,473 entries; version WBPS15 downloaded from parasite.wormbase.org in August 2020 (Tallon et al., 2014), assuming the digestion enzyme was trypsin with a maximum of 3 missed cleavages allowed. For timsTOF data, searches were performed with a fragment ion mass tolerance of 50 ppm and a parent ion tolerance of 25 ppm. For Q-Exactive data, searches were performed with a fragment ion mass tolerance of 20 ppm and a parent ion tolerance of 20 ppm. For all searches, carbamidomethylation of cysteine was specified in Peaks as a fixed modification. Deamidation of asparagine, formation of pyro-glutamic acid from N-terminal glutamine, acetylation of protein N-terminus, oxidation of methionine, and pyro-carbamidomethylation of N-terminal cysteine were specified as variable modifications. Peptide hits were cross-referenced against a human database (ENSEMBL for Human contaminants (Homo_sapiens.GRCh37.72 ENHU, version June 2016) to eliminate potential human peptides. Qualifying peptides had less than 1% false discovery rate and were absent from the human database.

We limited our list of detected proteins to those with at least one unique peptide (i.e. not shared with other *L. loa* or human proteins) detected with at least 2 mass features and a negative log P-value of 20 or higher (as defined by Peaks). We considered proteins detected in at least 80% of participant samples as potential cross-reactive biomarkers for loiasis. Each batch of participant samples included at least one endemic control and one non-endemic control (see study design). We excluded proteins if more than 10% of detected peptides matched human sequences, or if more than one matching peptide was detected in non-endemic control samples. To better illustrate the presence of one of more members of the Nas-14 family members across all tested samples, non-unique peptides (peptides from identical sequences across family members) were attributed to all family members, rather than only those for which a unique peptide was also detected.

### 2.9 Functional annotation of top *L. loa* biomarkers

Functional annotations were assigned to all Loa loa genes in the current genome annotation downloaded from WormBase Parasite (Howe et al., 2017) (PRJNA246086) using (i) Sma3s (version 2) (Casimiro-Soriguer et al., 2017), (ii) PANNZER (2022 release) (Törönen and Holm, 2022), (iii) KEGG gene annotations (Törönen and Holm, 2022) using GhostKOALA v2.2 (Kanehisa et al., 2016), and (iv) results from InterProScan v5.42 (Jones et al., 2014)to identify InterPro functional domains (Blum et al., 2021) and associated gene ontology classifications (The Gene Ontology Consortium, 2021). Potentially secreted proteins were identified using SignalP v5.0 (Almagro Armenteros et al., 2019) to identify signal peptides and to count transmembrane domains. For that analysis, proteins with fewer than 2 TM domains a predicted signal peptide were annotated as having a signal peptide, and proteins with 2 or more transmembrane domains were considered to be transmembrane proteins.

Protein conservation data across nematodes and hosts was quantified using orthologous protein family membership from OrthoFinder (v2.4.1) (Emms and Kelly, 2019), retrieved from Rosa et al., (2023). Additionally, the top BLAST (Altschul et al., 1990) (version 2.13.0+) hit for each *L. loa* protein to other species (Brugia malayi WS279 PRJNA10729.WBPS16, *Onchocerca volvulus* WS279 PRJEB513.WBPS16, *Wuchereria bancrofti* [locally improved annotation] and human GRCh38.106) was also identified, including the E value, alignment length, % identity, and whether the top hit was reciprocal (NCBI blastp v2.13.1+, default settings).

RNA-seq reads were retrieved from a previously published *Loa loa* dataset (Desjardins et al., 2013) and were mapped to the PRJNA246086 annotation of Loa loa using HiSat2 (v2.2.1), and reads were quantified per gene using featureCounts (from subread package v2.0.3). Stage-specific *Brugia malayi* gene expression data (PRJEB2709) was also mapped with the same approach to the *B. malayi* genome (PRJNA10729), and *B. malayi* genes were assigned to *L. loa* genes using the top BLAST hit. Relative gene expression in fragments per kilobase per million reads (FPKM) was calculated based on each gene’s length and the total number of reads mapped per sample.

### 2.10 Data analyses

Participant data collected in Cameroon (demographics, FTS, LoaScope, and CTBS results) were entered into a password protected REDCap database, and statistical analyses were performed using STATA (version. 17.0; StataCorp LLC., College Station, TX, USA). Figures were generated with Graph-Pad prism version 8. Categorical variables (such as gender, positivity to an assay) were summarized using frequencies and 95% confidence interval (CI) (Wilson, 1927). Continuous variables (such as age, Mf density, CFA levels, number of spectral counts) were described using median and interquartile range (IQR). The presence of detectable cross-reactive antigen at each follow-up time point was analyzed as a binary outcome and differences in the proportion of samples positive for cross-reactive antigen was compared using Chi-square test or Fischer exact test when applicable. Kappa statistic was used to determine the strength of agreement between the different CFA detecting assays. Finally, Mf density, antigen levels and peptide detected by MS were compared using Mann Whitney (FTS+ vs. FTS−) or Kruskal Wallis tests (for the different time point), while individual variations of the Mf density and CFA level were compared using Wilcoxon paired or Friedman tests. The cut-off for significance was set at 0.05 for all statistical analyses.

## 3 Results

### 3.1 Screening

Overall, 139 individuals aged 18–85 years (median: 49, IQR: 38–65) who were excluded from the TaNT study because of LoaScope Mf counts ≥20, 000Mf/mL were screened for inclusion in this study. One-hundred-thirty-four (96.4%) were microfilaremic for *L. loa*, with Mf counts ranging from 20–114,240 Mf/mL of blood (Median: 22,780; IQR: 9,280–46,155). Eight (5.8%) of the screened individuals were also microfilaremic for *Mansonella perstans*. None were positive for *W. bancrofti* by either nocturnal thick blood smear or qPCR. We did not test for onchocerciasis; we felt that the low likelihood of detecting circulating *Onchocerca volvulus* antigens did not justify subjecting study participants to skin snips.

### 3.2 Enrolment and follow-up

Seventy-three of the screened individuals (52.5%) were FTS positive, all of whom were enrolled, along with 13 FTS-negative controls having *L. loa* Mf counts >20,000/mL (Figure 1). Enrollees were aged 18 to 80 years old (median: 50; IQR: 38–59) and approximately one third (35%) were females.

Among participants who were FTS positive, (78.1%) had a semiquantitative FTS score of 1, 15 (20.5%) had an FTS score of 2, and 1 (1.4%) an FTS score of 3. *L. loa* Mf counts of FTS-positive enrollees ranged from 6,680 to 114,240 (Median: 34,450; IQR 22,860–54,720) while *L. loa* Mf counts of FTS negative controls ranged from 21,780 to 98,120 (Median: 42,480; IQR 26,260–49,980) with no statistically significant difference between the two groups (Mann Whitney U: 378; p-value: 0.2494).

To determine whether cross-reactive antigenemia is transient or stable, we followed individuals over time. Over the course of the study, 18 participants were lost to follow-up; 10 (11.6%) withdrew from the study, six (6.9%) moved outside the study area, and two (2.3%) passed away. During follow-up, approximately 75% were present at 6, 9, and 12-months, and 64% were seen at 15-months follow-up. Fifty-eight (67.4%) participants were present during all study visits.

### 3.3 *L. loa* Mf counts and FTS status remains constant over 15-months

**FIGURE 2:**
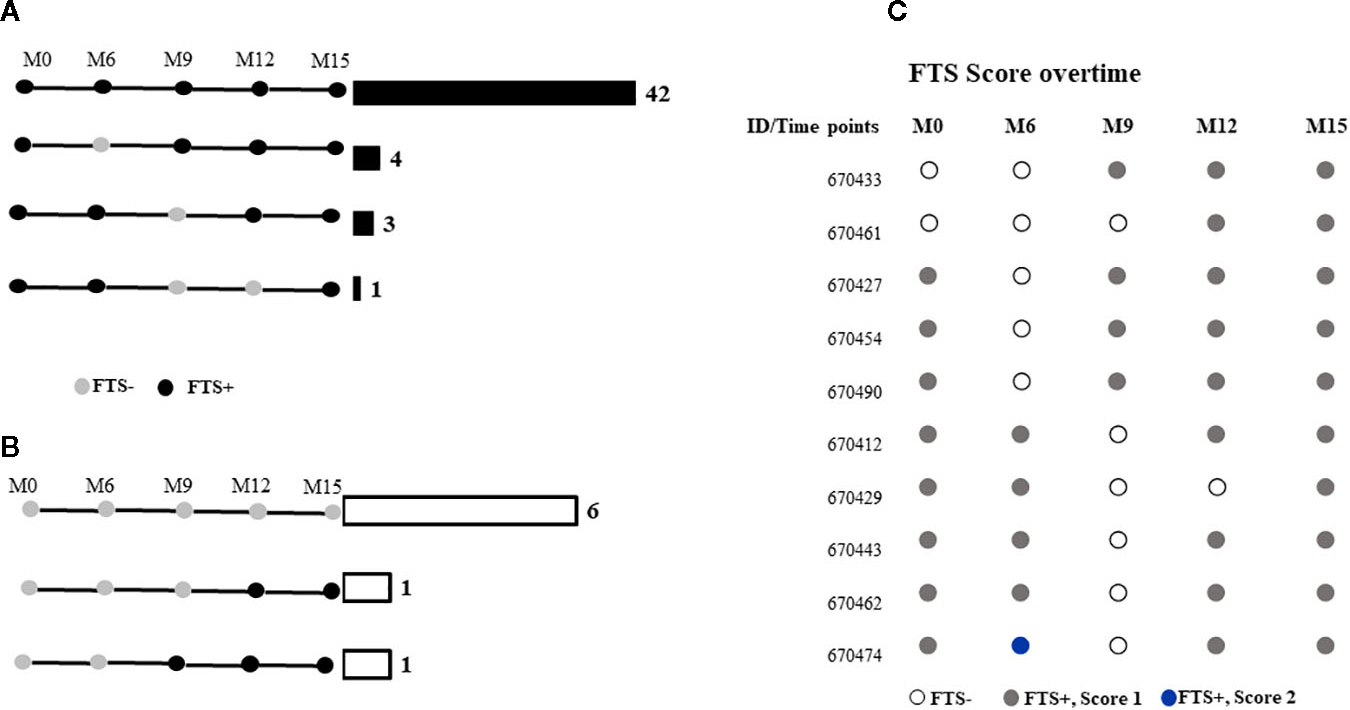
FTS status of participants at months 0, 6-, 9-, 12-, and 15-months post-enrolment among those who were **(A)** FTS-positive at baseline, or **(B)** FTS-negative at baseline. Horizontal bars and numbers indicate the number of individuals exhibiting the indicated pattern of antigenemia. **(C)** Semiquantitative FTS scores over time among participants whose status changed over the course of the study.

Among the 58 participants with data at all the time points, 48 (82.8%) did not have a change in FTS status during the study, while 10 (17.2%) experienced at least one FTS conversion event over time. All individuals who experienced FTS conversion events at some point in the study had a semiquantitative FTS score of one at baseline (Figure 2).

**FIGURE 3:**
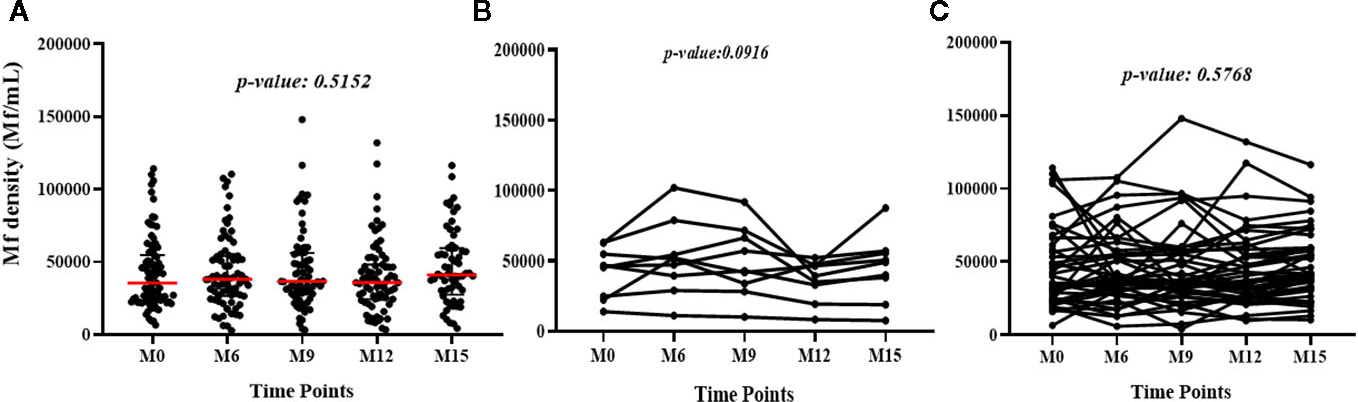
Variation of *L. loa* Mf counts over time among **(A)** all participants for all the time points (The p-value shows the comparison of the median Mf count between time points using the Kruskal Wallis test), **(B)** individuals who experienced FTS status changes (the p-value shows the comparison of individual Mf count over time using Friedman test) and **(C)** individuals with constant FTS over time (the p-value shows the comparison of individual Mf count over time using Friedman test).

Median *L. loa* Mf densities were stable over time in the study population as a whole, and among the subset of individuals who conserved their FTS status over the course of the study, or among those who experienced at least one FTS conversion event (Figure 3).

### 3.4 Quantitative antigen levels

**FIGURE 4:**
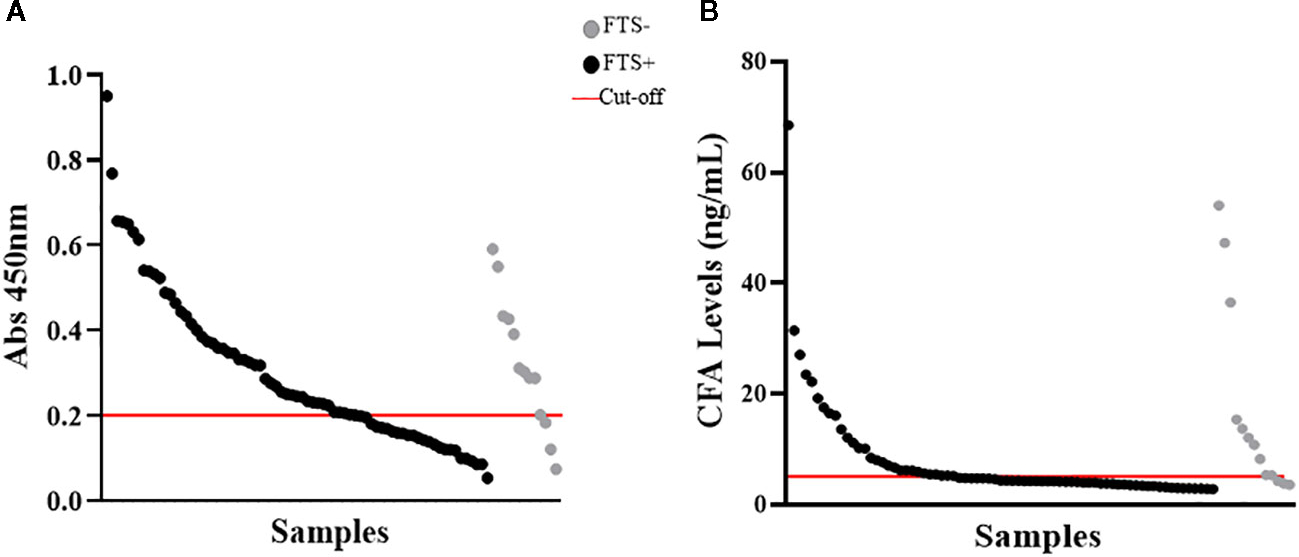
Detection of cross-reactive loiasis antigens by ELISAs at baseline. The x-axis shows the samples (each dot represents a sample) and the y-axis represents the OD in **(A)** and the cross-reactive antigen concentration in **(B). (A)** shows the detection of cross-reactive antigens by Og4C3 ELISA and **(B)** shows the detection of cross-reactive antigens by in-house circulating filarial antigen (CFA) ELISA. In both panels the grey color represents individuals with negative Filariasis Test Strip (FTS) results (n=13), the black color represents individuals with positive FTS results (n=73), and the red line is the cut-off of positivity (set at an antigen level of 6ng for the CFA ELISA and at an OD of 0.2 for the Og4C3 ELISA).

To quantify baseline cross-reactive antigen levels, we used the commercially available TropBio Og4C3 ELISA and an in-house CFA ELISA. There was minimal agreement (kappa score =0.335) between these assays and the FTS; CFA positivity was 35% by in-house ELISA and 69% Og4C3 ELISA (Figure 4). Quantitative CFA ELISA results varied significantly over time (Chi-square: 11.166; p-value: 0.0248; see Table 2).

### 3.5 Western blot results

**FIGURE 5:**
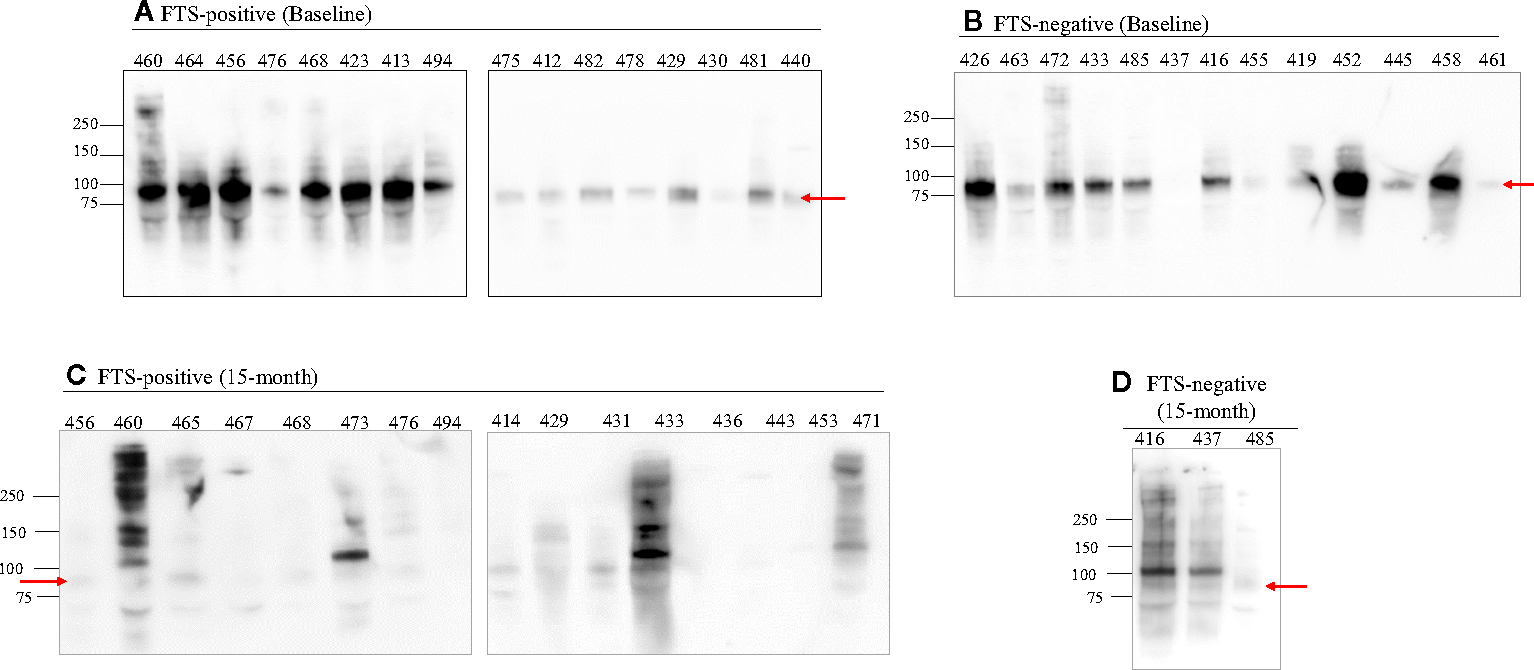
An 80 kDa antigen in predominant in FTS positive and FTS negative loiasis samples. Representative western blots of FTS-positive **(A, C)** and FTS-negative **(B, D)** samples show the presence of multiple cross-reactive antigens, including a predominant band at 80kDa (arrow). For each sample, 1mL of serum was immunoprecipitated with DH6.5 antibody-conjugated beads to capture cross-reactive antigens. The proteins were resolved by SDS-PAGE and immunoblotted with AD12-HRP. An abbreviated sample identifier is written above each lane. All baseline (n=86) samples and 38 month 15 samples (30 FTS+ and 9 FTS−) were tested by western blot; representative examples are shown. Although not represented on the figure, the molecular weight corresponding to each detected band was determined using Bio-Rad Precision Plus Protein Standards.

To better characterize the cross-reactive antigens, cross-reactive antigens from all baseline samples (73 FTS-positive and 13 FTS-negative controls) and a portion of the 15-month samples ( 30 FTS-positive and 8 FTS-negative controls) were immunoprecipitated and detected by western blot. Seventy-nine of the baseline samples (91.9%) and 33/38 15-month samples had at least one reactive protein band by western blot. Notably, a predominant ~80kDa antigen was detected in all positive baseline samples. This ~80kDa antigen was less well detected in the 15-month samples (Figure 5).

### 3.6 Agreement between antigen detection methods

No agreement was found between FTS status, CFA ELISA, Og4C3 ELISA, and western blot. Indeed, kappa analysis showed no agreement between FTS and CFA ELISA (Kappa score: −0.1331) or Og4C3 ELISA (Kappa score: −0.0629), nor between western blot and FTS (Kappa score: −0.0079), CFA ELISA (Kappa score: −0.0556), and Og4C3 ELISA (Kappa score: -−0.0098). There was minimal agreement was found between the two ELISA formats (Kappa score: 0.2609) (Figure S1).

### 3.7 Mass spectrometry analysis of potential cross-reactive biomarkers

**FIGURE 6:**
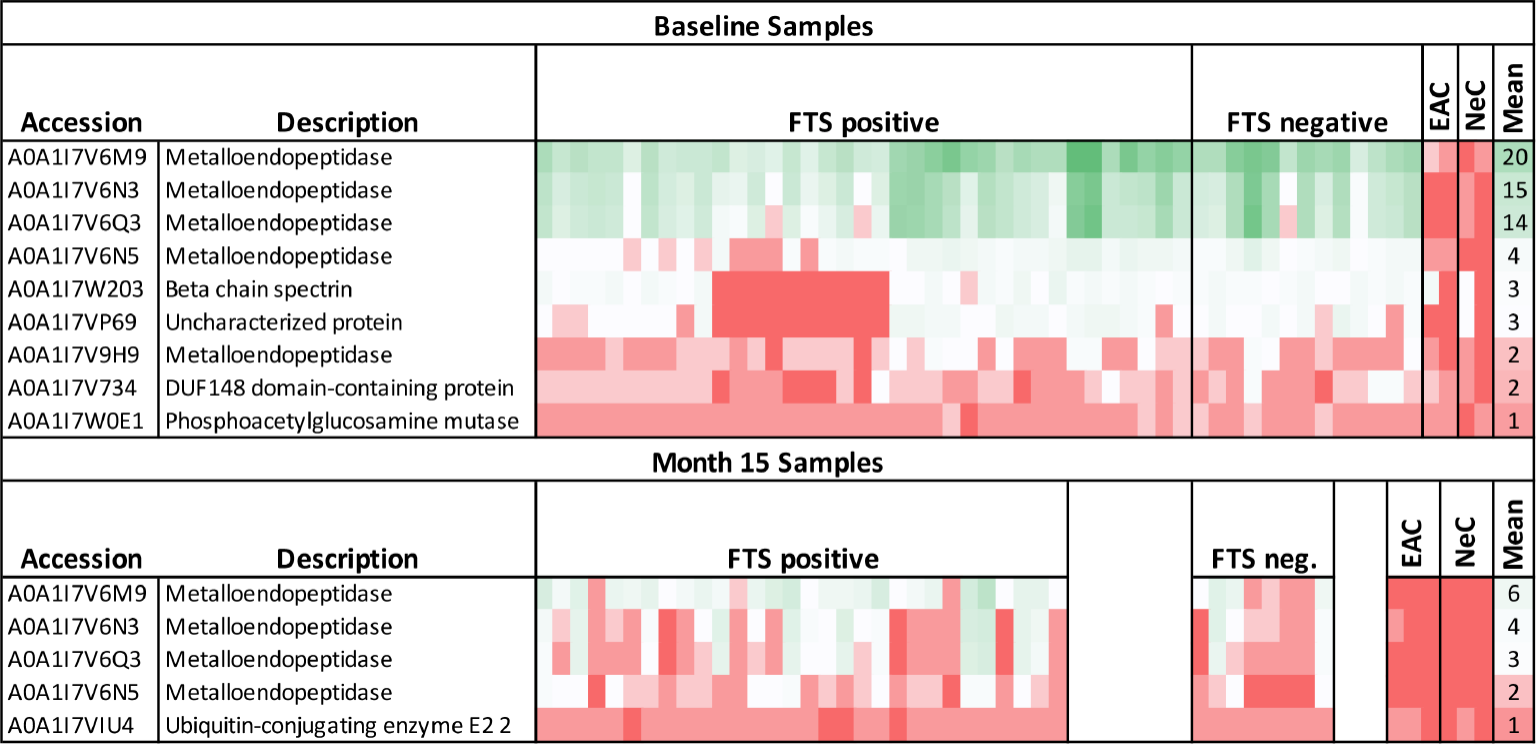
Heatmap of mass spectrometry detection of *Loa loa* proteins representing protein abundances. The columns represent baseline plasma samples from 50 individual participants, grouped by FTS status (37 FTS-positive and 13 FTS-negative), two endemic, amicrofilaremic controls (EAC), and two non-endemic controls (NeC). Rows represent individual proteins detected, and numbers indicate the number of peptides for each protein detected in each individual sample. The mean number of peptides detected among loiasis samples is indicated as an approximate guide to the color scheme. All the metalloendopeptidase represented here belong to the Nas-14 protein family.

To identify potential protein biomarkers of cross-reactive loiasis, we used liquid chromatography with tandem mass spectrometry (LC-MS/MS) to analyze immunoprecipitated proteins from 50 baseline samples (37 FTS-positive and 13 FTS-negative) and 38 15-month samples (30 FTS-positive and 8 FTS-negative). Fifty-six *L*. *loa* proteins were detected by MS in 50% or more of baseline participant samples (Table S1), and seven proteins met this criterion at 15-months (Table S2). Only nine proteins met our prespecified threshold of detection in at least 80% of loiasis sera (Figure 6); five of these were Nas-14 family metalloproteases. Sequences for all detected proteins are provided are available and posted on a data repository. Unexpectedly, the top biomarker candidates were detected similarly among FTS-positive and FTS-negative loiasis samples (Tables S1, S2).

Among baseline samples, a significant correlation was found between Mf counts and the number of *L. loa* peptides detected per sample (Spearman rho: 0.4336; p-value: 0.0017), but not between the number of *L. loa* peptides detected per sample and CFA levels measured by in-house ELISA (rho: 0. 0.2508; p-value: 0. 0.0776). For 15-month samples, both Mf density (rho: 0.3832; p-value: 0.0176) and CFA levels (rho: 0.5014; p-value: 0.0013) were correlated with the number of *L. loa* peptides detected per sample (Figure S2).

Predicted characteristics available via bioinformatics databases for the top ten biomarkers are provided in a Supplemental Table (Table S3). The Nas-14 protease most abundantly detected is a zinc metalloprotease predicted to be secreted, without transmembrane domains. Its predicted percent identity to the closest homologue in other filarial species is 46.5% for *Brugia malayi*, 52.5% for *Onchocerca volvulus*, and 59.4% for *W. bancrofti.* Interestingly, one of the Nas-14 homologues detected is predicted among the top 3% of expressed *L. loa* proteins (Table S3).

## 4 Discussion

FTS cross-reactivity in individuals with loiasis represents an obstacle to LF mapping in loiasis-endemic areas. Although loiasis cross-reactivity with LF-RDTs is associated with high *L. loa* Mf density, it is unclear why only some heavily infected persons are cross-reactive and furthermore why cross-reactive antigenemia is transient in some individuals. This study aimed to investigate the stability of loiasis cross-reactivity over time and determine which *L. loa* antigens are consistently present in cross-reactive sera. To the best of our knowledge, this study is the largest examination of loiasis cross-reactive antigenemia to date and to examine its persistence over time.

We found that FTS cross-reactivity is relatively stable among individuals with very high *L. loa* Mf counts over a short time (15 months). Although a proportion of participants experienced FTS status changes from one visit to another, most maintained the same Mf status throughout the study. Among those who did experience FTS conversion events, the changes were from weakly positive (1+) to negative or vice versa; and as observed previously, changes in FTS status were not associated with variation in *L. loa* Mf density (Hertz et al., 2018). These data suggest that FTS status changes are more likely among individuals with antigen levels near the limit of detection, a pattern more consistent with fluctuations in relatively steady state of antigenemia than with intermittent spikes of antigen release, as previously hypothesized (Hertz et al., 2018). Whether this fluctuation in antigen level is modulated by antigen release from Mf or adult worms, or by fluctuations in antigen clearance remains unclear and represents an avenue for future research. Interestingly, in the month 15 samples the number detected peptides was lower and the western blot patterns were more variable. The reasons for this are unclear but might include seasonal variability in antigen release or clearance. Further studies would be required to test this hypothesis.

Each of the assays used to assess antigenemia in this study (the two ELISA formats, FTS, and AD12.1-HRP western blot) detects filarial antigens by binding to the same carbohydrate moiety (the AD12.1 epitope) (Weil et al., 1987), yet there was little to no agreement between these assays. The reasons for this are unclear but may arise from multiple differences in test formats and reagents. The Og4C3 monoclonal antibody, for example, binds to a broader subset of carbohydrates on a glycan array than the AD12.1 monoclonal antibody (used in the FTS and the in-house ELISA) (Hertz et al., 2020), which may result in recognition of a wider variety of cross-reactive *L. loa* antigens. Disagreement between the ELISA and FTS may be due to differing availability of cross-reactive epitopes. In the FTS, AD12.1 is immobilized on a nitrocellulose membrane to capture antigens bound to gold-labeled polyclonal anti-filarial antisera, whereas the CFA ELISA uses the analogous antibodies (DH6.5 and AD12.1 recognizing the same carbohydrate moiety) to both capture and detect, theoretically limiting detection to antigens with multiple glycan epitopes. Immunoprecipitation and western blot, although using the same reagents, are not subject to the same limitation since immune complexes formed between antigen and DH6.5 are dissociated after immunoprecipitation making all the available epitopes free for AD12.1 detection during western blot. Additionally, FTS uses a small sample volume (70 μl of fresh whole blood), while the ELISA assays were performed on thawed plasma samples with an intermediate heating step that may alter which epitopes are available for antibody binding. In addition, proteins detected by western blot were concentrated by immunoprecipitation. These differences in detection by different assays underscore our prior observation that *L. loa* cross-reactive antigenemia is fundamentally different than *W. bancrofti* antigenemia. While *W. bancrofti* CFA is a 200+ kDa heat stable glycoprotein containing multiple AD12.1 epitopes (Weil et al., 1987), loiasis cross-reactive antigens are a mixture of glycoproteins that may vary from one individual to another or even within a given individual over time.

An important outcome of this study is the identification of promising candidates for potential loiasis antigen detection assays. Nas-14 family metalloproteases were detected in nearly all loiasis samples by MS. Furthermore, a yet unidentified 80kDa antigen was detectable by western blot in nearly all tested samples, regardless of FTS status. It is not clear whether the 80kDa band is a Nas14 metalloprotease, but this seems unlikely; the predicted molecular weight of Nas-14 is ~37 kDa and we found no relationship between Nas-14 presence/abundance and positivity to western blot.

One of our goals in conducting this study was to identify one or more *L. loa* proteins consistently present in loiasis-infected individuals over time. Such a molecule (or molecules) may prove useful for two reasons. First, if cross-reactive antigens prove to be present in all individuals with loiasis, but at levels not detected by the FTS, a more sensitive assay for such antigens could function as an antigen detection assay for loiasis. Such an assay could improve mapping and clinical monitoring of loiasis. Second, an assay specific for cross-reactive *Loa* antigens would aid LF elimination programs. Although a positive test for cross-reactive *Loa* antigens would not rule out the possibility of *W. bancrofti* co-infection, at the population level the absence of any FTS positive individuals not also positive for *Loa* antigens would suggest that bancroftian filariasis was rare or absent.

One important limitation of our study was the inclusion of only individuals with very high *L. loa* Mf counts. It was necessary to screen heavily infected individuals to identify a large cohort of individuals with cross-reactive antigenemia. This also provided an optimal population for biomarker discovery, but our results may not be typical for individuals with lower microfilarial loads. A second limitation is our inability to definitively exclude co-infection with other filarial infections. Although we tested for blood-borne microfilariae (*L. loa*, *W. bancrofti* and *M. perstans*) by thick blood smear, and ruled out *W. bancrofti* coinfection by PCR, we did not test for *O. volvulus* However, it is unlikely that our results significantly impacted by *O. volvulus* co-infection. The study was conducted in a hypo-endemic area for onchocerciasis with Mf prevalence is less than 18% (Kamgno et al., unpublished data) and there is no prior evidence to suggest detection of cross-reactive antigens in individuals with onchocerciasis. Another potential limitation relative to biomarker discovery i s that our discovery approach relied on immunoprecipitation with the DH6.5 antibody. In theory, this should capture cross-reactive antigens containing the AD12.1 carbohydrate epitope, yet many antigens captured previously by this method are not predicted to be targets for glycosylation (Hertz et al., 2018). It is possible that our method of immunoprecipitation using the DH6.5 antibody captures non-cross-reactive antigens lacking the AD12.1 carbohydrate that may be complexed with AD12.1-epitope containing cross-reactive antigens.

In conclusion, this study has shown that in individuals with high *L. loa* Mf counts, FTS status changes occur primarily among individuals with borderline FTS results. Our data confirm that in a large cohort of individuals, multiple *L. loa* cross-reactive antigens are present in both FTS positive and FTS negative individuals. Finally, this study has identified one or more loiasis proteins that are consistently detected in individuals with heavy loiasis infections. Future work to develop assays using these biomarkers should lead to improved antigen detection tests capable of distinguishing loiasis from lymphatic filariasis.

## Supplementary Material

Table S1 SUPPLEMENTARY TABLE 1
Heatmap of mass spectrometry detection of *Loa loa* proteins representing protein abundances for baseline samples.

Image S1 SUPPLEMENTARY FIGURE 1
Comparison between the different AD12 epitope detecting assays. **(A)** Compare FTS and IH sandwich ELISA. **(B)** Compare FTS and TropBio ELISA. **(C)** Compare the two ELISA formats. **(D)** Compare FTS and western blot. **(E)** Compare TropBio ELISA and western blot. **(F)** Compare IH sandwich ELISA and western blot.

Image S2 SUPPLEMENTARY FIGURE 2
Correlation between the number of MS peptides, Mf density and antigen levels. **(A)** Shows the correlation between the number of MS peptides and Mf density at baseline. **(B)** Shows the correlation between the number of MS peptide antigen level determined by IH sandwich ELISA at baseline. **(C)** Shows the correlation between the number of MS peptides and Mf density at 15-month. **(B)** Shows the correlation between the number of MS peptide antigen level determined by IH sandwich ELISA at 15-month.

Table S2 SUPPLEMENTARY TABLE 2
Heatmap of mass spectrometry detection of *Loa loa* proteins representing protein abundances for 15-months samples.

Table S3 SUPPLEMENTARY TABLE 3
Predicted characteristics available via bioinformatics databases for the top ten biomarkers.

## Figures and Tables

**TABLE 1:** Antibodies used to capture cross-reactive antigens.

Antibody	Origin	Main targeted antigen	Known Cross-reactivity	Test used
AD12.1 (Weil et al., 1987)	Monoclonal IgM against *Dirofilaria immitis*	- Recognizes a ~200kDa glycoprotein of W. bancrofti present in patient sera. - Multiple carbohydrate antigens of other filarial and non-filarial nematode, but that are not expected to be present infected sera	Yes (*L. loa*)	- FTS - In-house ELISA - AD12.1-Western blot
DH6.5 (Weil et al., 1987)	Monoclonal IgM against *Dirofilaria immitis* Analog of AD12.1.1	- Recognize a ~200kDa glycoprotein of W. bancrofti present in patient sera. - Multiple carbohydrate antigens of other filarial and non-filarial nematode, but that are not expected to be present infected sera	Yes *(L. loa)*	- In-house CFA ELISA - AD12.1-Western blot
Og4C3 (More and Copeman, 1990)	Monoclonal IgM against *Onchocerca gibsoni*	- Binds to a broader subset of carbohydrates Ag (molecular weight > 130 kDa and between 50–60 kDa) - Can recognize antigen for a wide range of filarial and non-filarial nematode	Yes (*L. loa*)	TropBio Og4C3 ELISA

**TABLE 2:** Variation of CFA level over time.

	In-house ELISA (Level in ng/mL)			Og4C3 ELISA (In OD unit)		
	Negative	Positive(%)	Range (median)	Negative	Positive(%)	Range (median)
Baseline	56	30(34.9)	0.78–68.54 (4.74)	26	59(69)	0.045–0.95(0.25)
M6	45	31(40.8)	0.78–97.04(4.07)			
M9	39	36(48)	0.78–154.55(5.071)	–	–	–
M12	45	30(40)	0.78–114.88(4.66)	–	–	–
M15	28	36(56.3)	0.78–179.35(7.56)	–	–	–

## Data Availability

The original contributions presented in the study are included in the article/Supplementary Files, further inquiries can be directed to the corresponding author/s.
