# Supplementary figures and images for: Longitudinal study of cross-reactive antigenemia in individuals with high Loa loa microfilarial density reveals promising biomarkers for distinguishing lymphatic filariasis from loiasis
Source: Front Parasitol. 2023 Nov 17;2:1292837. doi: 10.3389/fpara.2023.1292837 (PMC11340908; doi:10.3389/fpara.2023.1292837)

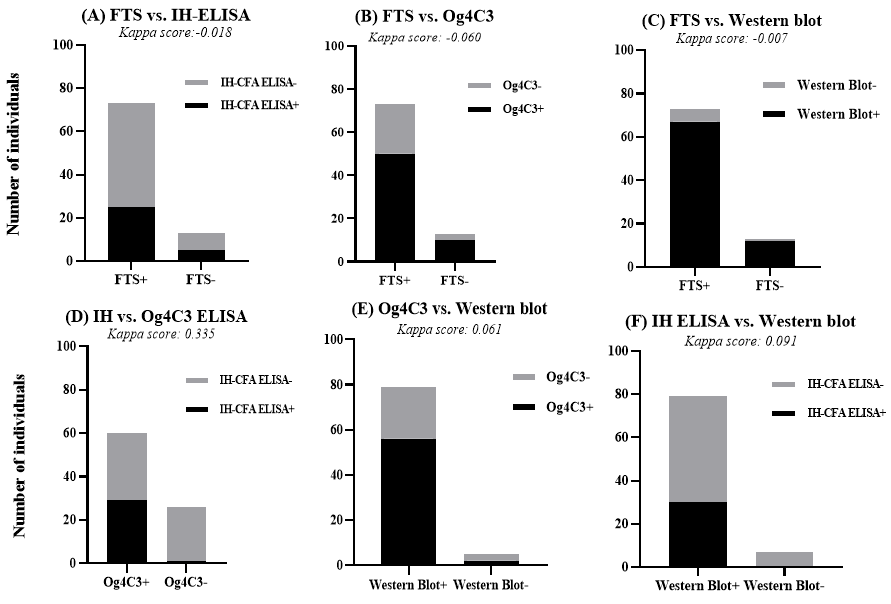

Supplement: Supplementary Figure 1 — Comparison between the different AD12 epitope detecting assays. (A) Compare FTS and IH sandwich ELISA. (B) Compare FTS and TropBio ELISA. (C) Compare the two ELISA formats. (D) Compare FTS and western blot. (E) Compare TropBio ELISA and western blot. (F) Compare IH sandwich ELISA and western blot. [file Image_1.tif]

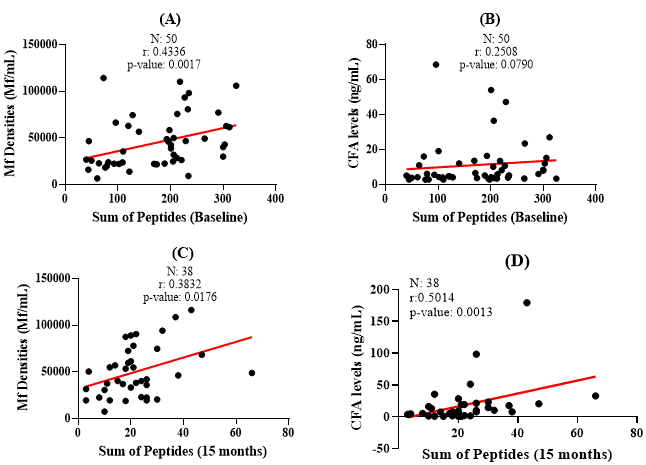

Supplement: Supplementary Figure 2 — Correlation between the number of MS peptides, Mf density and antigen levels. (A) Shows the correlation between the number of MS peptides and Mf density at baseline. (B) Shows the correlation between the number of MS peptide antigen level determined by IH sandwich ELISA at baseline. (C) Shows the correlation between the number of MS peptides and Mf density at 15-month. (B) Shows the correlation between the number of MS peptide antigen level determined by IH sandwich ELISA at 15-month. [file Image_2.tif]
